# A Novel Hypoxia Related Marker in Blood Link to Aid Diagnosis and Therapy in Osteoarthritis

**DOI:** 10.3390/genes13091501

**Published:** 2022-08-23

**Authors:** Shunhan Yao, Meiling Deng, Xiaojing Du, Rongzhi Huang, Qingfeng Chen

**Affiliations:** 1 Medical College, Guangxi University, Nanning 530000, China; 2 School of Information and Management, Guangxi Medical University, Nanning 530000, China; 3 School of Computer, Electronic and Information, Guangxi University, Nanning 530000, China; 4 Traumatic Orthopedic Hand Surgery, The First People’s Hospital of Qinzhou, Qinzhou 535000, China

**Keywords:** osteoarthritis, hypoxia, novel hypoxia-related index, immune microenvironment

## Abstract

Osteoarthritis (OA) is a common chronic degenerative arthritis. Its treatment options are very limited. At present, hypoxia is a prominent factor in OA. This study aimed to re-explore the mechanism between hypoxia and OA, which provides new insights into the diagnosis and therapy of OA. We acquired the OA-related expression profiles of GSE48556, GSE55235, and GSE55457 for our analysis. Using gene set variation analysis (GSVA), we found significant differences in hypoxia. These differences result from multiple pathways, such as the p53 signaling pathway, cell senescence, the NF-kappa B signaling pathway, Ubiquitin-mediated proteolysis, and apoptosis. Meanwhile, the single-sample gene set enrichment analysis (ssGSEA) showed that hypoxia was significantly associated with the level of immune cell infiltration in the immune microenvironment. Thus, we believe that hypoxia is useful for the diagnosis and treatment of OA. We successfully constructed a novel hypoxia-related index (HRI) based on seven hypoxia-related genes (ADM, CDKN3, ENO1, NDRG1, PGAM1, SLC2A1, VEGFA) by least absolute shrinkage and binary logistic regression of the generalized linear regression. HRI showed potential for improving OA diagnosis through receiver operation characteristic (ROC) analysis (AUC training cohort = 0.919, AUC testing cohort = 0.985). Moreover, we found that celastrol, droxinostat, torin-2, and narciclasine may be potential therapeutic compounds for OA based on the Connectivity Map (CMap). In conclusion, hypoxia is involved in the development and progression of OA. HRI can improve diagnosis and show great potential in clinical application. Celastrol, droxinostat, torin-2, and narciclasine may be potential compounds for the treatment of OA patients.

## 1. Introduction

Osteoarthritis (OA) is one of the most common chronic degenerative arthritis in the aged. One-third of people over the age of 65 suffer from OA [1]. The prevalence of osteoarthritis is increasing as the global population ages and obesity increases [2]. Due to the lack of effective treatment, the severe pain and irreversible cartilage damage associated with osteoarthritis greatly reduce the patient’s quality of life [3]. Therefore, it is essential to explore the pathogenesis of OA and predict some new drugs, to improve the treatment and quality of life of OA patients.

In recent years, OA has mainly been characterized by articular cartilage degradation, varying degrees of synovial inflammation, subchondral bone remodeling, and osteophyte formation, leading to pain and a loss of joint function [4,5]. Studies have shown that it is related to joint cavity factors such as age, weight, genetics, immune factors, biomechanical changes, intestinal microbiota [6], cellular and matrix metabolic disorders, signaling pathways, and cytokines [7,8]. Recent studies have found that an anoxic environment plays an important role in osteoarthritis, especially knee arthritis [9]. Under the condition of pathological hypoxia, the body produces a hypoxia-inducible factor that affects cartilage synovial bone metabolism [10,11]. Zhou et al. found that hypoxia can induce the expression of catabolic factors in fibrocystic-like synovial cells and enhance the role of inflammatory factors, which are involved in the occurrence and development of OA [12]. Ryu et al. confirmed that hypoxia could enhance Fas expression and activate downstream signaling pathways, promote chondrocyte apoptosis and autophagy, and lead to cartilage destruction in OA [13]. Thus, understanding and exploring the hypoxia environment is of great value for revealing the pathogenesis and clinical diagnosis of OA.

In this study, we estimated the hypoxias status of the samples using gene set variation analysis based on the levels of 15 gene expression signatures (ACOT7, ADM, ALDOA, CDKN3, ENO1, LDHA, MIF, MRPS17, NDRG1, P4HA1, PGAM1, SLC2A1, TPI1, TUBB6, and VEGFA). Studies have documented that these genes performed the best when classifying hypoxia status [14,15]. Next, we analyzed the hypoxia-related pathway and the immune microenvironment to explore the possible mechanism of OA. We further established a new maker composed of hypoxia genes to improve the diagnosis of OA. Finally, we screened potential drugs related to hypoxia therapy of OA. Our results provided new insights into the pathogenesis and treatment of OA.

## 2. Materials and Methods

### 2.1. Data Pre-Processing

We acquired the OA gene expression profile of GSE48556, GSE55235, and GSE55457 from Gene Expression Omnibus (GEO, http://www.ncbi.nlm.nih.gov/geo/) (accessed on 25 March 2022) [16] using the GEOquery R package. The raw datasets of GSE55235 and GSE55457 were further processed for background correction, normalized with the RMA algorithm, and removed batch effects using the “affy” R package. The data of GSE48556 as a training cohort were normalized through the “limma” R package [17]. Next, the sva algorithm was performed to merge the GSE55235 and GSE55457 datasets for the testing cohort and remove the batch effect. Finally, the probe IDs were transformed into gene symbols using corresponding R packages (Table 1). Gene symbols with multiple probe IDs retained their mean expression.

### 2.2. Assessment of Hypoxia Status by Gene Set Variation Analysis

The hypoxia score of each sample of GSE48556 was calculated by gene set variation analysis (GSVA) based on 15 hypoxia gene expression signatures (ACOT7, ADM, ALDOA, CDKN3, ENO1, LDHA, MIF, MRPS17, NDRG1, P4HA1, PGAM1, SLC2A1, TPI1, TUBB6, and VEGFA) [18,19]. A *t*-test was used to determine the differential hypoxia status between osteoarthritis and the normal samples. *p* < 0.05 were considered statistically significant.

### 2.3. Classification of Hypoxia Status in OA

Two different hypoxia status groups were classified among 106 OA samples using the “ConsensusClusterPlus” package in R software (Version 4.0.4) [20]. Euclidean distance was used to calculate the similarity among samples, while K-means was used for clustering. We then performed 50 iterations with a resampling rate of 0.8. Using the cumulative distribution function (CDF), we figured out the optimal number of clusters. Hypoxia subtypes were verified through principal component analysis (PCA). The difference in the hypoxia genes and the hypoxia score between the two clusters were identified by *t*-test analysis.

### 2.4. Functional Enrichment Analysis

To explore the potential mechanism of hypoxia in OA, we performed a Kyoto Encyclopedia of Genes and Genomes (KEGG) pathway analysis to identify key signaling pathways [21,22]. Before analysis, an ordered gene list was generated using the edgeR package, and genes with *p* < 0.05 and |r| > 0.6 were reserved for this analysis.

### 2.5. Immunity Analysis

With the deepening of the OA research, a large number of studies report that OA was related to individual immunity [23,24]. In order to explore the mechanism of hypoxia, we analyzed the relationship between hypoxia and the immune microenvironment. Firstly, we assessed 29 immune cell levels by single-sample gene set enrichment analysis (ssGSEA) based on 29 immune gene sets (Supplementary Table S1). Subsequently, we evaluated the correlation between 29 immune cells and hypoxia score. The immune cells with *p* < 0.05 and |r| > 0.3 were identified as significantly correlated with hypoxia. Meanwhile, we used a *t*-test to identify differential immune cells between the clusters.

### 2.6. Construction and Verification of Hypoxia-Related Diagnostic Marker in Blood Link

Previous studies have revealed that hypoxia was highly associated with OA [25,26]. We also found that hypoxia played an essential role in OA in our above analysis. Therefore, we tried to use hypoxia-related genes to construct hypoxia-related markers for improving the diagnosis of OA. First of all, the least absolute shrinkage and selection operator (LASSO) regression analysis was used to select important genes for hypoxia-related markers [27]. Further, a hypoxia-related index (HRI) was constructed as a novel marker using logistic regression of generalized linear regression (GLM) algorithm. Finally, we used an independent cohort to verify the potentiality and accuracy of HRI.

### 2.7. Connectivity Map Analysis

At present, there is still a lack of effective treatment that can prevent or delay OA progression [28]. A new approach for solving this serious problem is by exploring drugs aimed at hypoxia status for the treatment of OA. Thus, we used Connectivity Map (CMap, http://www.broadinstitute.org/cmap/) (accessed on 25 March 2022) to detect new potential drugs for OA treatment [29]. Then, we performed a Pearson correlation analysis to calculate the relationship between each gene and hypoxia score. The genes with a top 150 positive correlation with the hypoxia score were up-regulated genes for input, while the genes with a top 150 negative correlation with the hypoxia score were the down-regulated genes for input. The compounds with an absolute value of connective score larger than 98.8 were identified as potential therapeutics. 

### 2.8. Statistical Analysis

All of the statistical analyses were performed using the R software (version 4.0.4) (http://www.r-project.org/) (accessed on 26 March 2022) and the corresponding R packages. *p* < 0.05 indicated statistically significant differences in this analysis. A T-test was used to evaluate the statistical differences between the high and the low hypoxia score, as well as the differences in the clinical features and the gene expression levels between the normal and OA samples. The relationship between the hypoxia score and the gene expression level was evaluated through a Pearson correlation analysis. The Area Under Curve (AUC) of the receiver operating characteristic (ROC) curve was calculated using the ROCR R package.

## 3. Results

### 3.1. Hypoxia Status Played Crucial Role in OA

**Figure 1:**
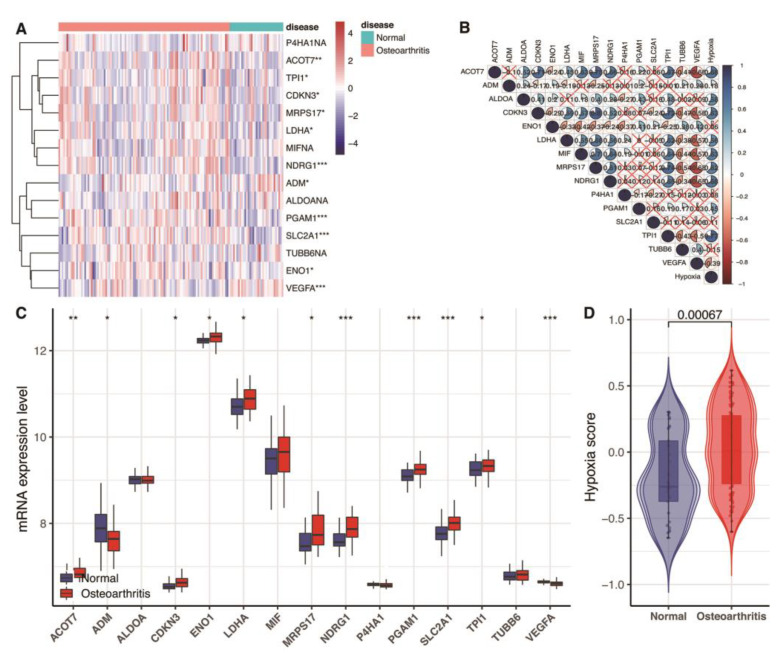
Assessment of hypoxia status in GSE48556. (**A**) Heat map of the expression of 15 hypoxia gene expression signatures. Red represents high expression, and blue represents low expression. (**B**) The correlation between the 15 hypoxia gene expression signatures and hypoxia score. Blue represents positive correlation; red represents negative correlation. (**C**) Box plots show 15 differentially expressed hypoxia gene expression signatures. * *p* < 0.05; ** *p* < 0.01; *** *p* < 0.001; *p* ≥ 0.05, not significant. (**D**) Violin graphs of hypoxia scores between osteoarthritis and normal samples.

In this study, we found nine up-regulated genes and two down-regulated genes in 15 hypoxia gene expression signatures in the blood link of OA (Figure 1A,C). Further, we observed that the hypoxia score exhibited an obvious statistical difference between the normal and OA samples (Figure 1D, *p* < 0.05). These hypoxia-related genes and hypoxia correlated with each other (Figure 1B).

### 3.2. Classification of Hypoxia Status

**Figure 2:**
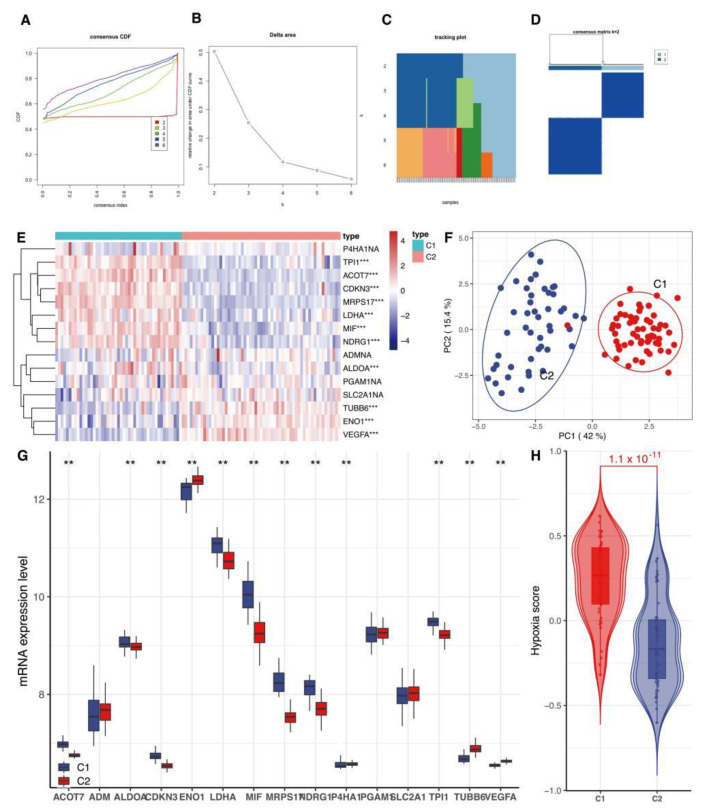
Classification of hypoxia status. (**A**) CDF delta area curve of consensus clustering, indicating the relative change in area under the cumulative distribution function. CDF curve for each category number k compared with k − 1. The horizontal axis represents the category number k, and the vertical axis represents the relative change in area under CDF curve. (**B**) CDF curve; different colors reflect different cluster numbers, the horizontal axis represents the consensus index, the vertical axis stands for CDF. (**C**,**D**) Heatmap of sample clustering at consensus K = 2. (**E**) Heat map of the expression of 15 hypoxia gene expression signatures between cluster1 (C1) and cluster2 (C2). (**F**) PCA analysis of two subtypes. PCA: principal component analysis. (**G**) Box plots show differentially expressed 15 hypoxia gene expression signatures between C1 and C2. ** means *p* < 0.01, *** *p* < 0.001, ns means not statistically significant. (**H**) Violin graphs of hypoxia scores between C1 and C2. CDF: cumulative distribution function.

Our analysis found the flattest middle segment of the CDF curve when K = 2 in the CDF curve (Figure 2A). In addition, the interference between subtypes could be reduced to a minimum when K = 2 was selected for the consensus clustering analysis (Figure 2B–D). Therefore, two hypoxia subtypes: cluster1 (C1) and cluster2 (C2), were identified. The result showed that the two clusters exhibited a statistically significant difference (Figure 2H, *p* < 0.05). Our clustering result was verified by PCA analysis (Figure 2F). Many hypoxia-related genes exhibited a significant distinction between the two subtypes (Figure 2E). 

### 3.3. Functional Enrichment Analysis

**Figure 3:**
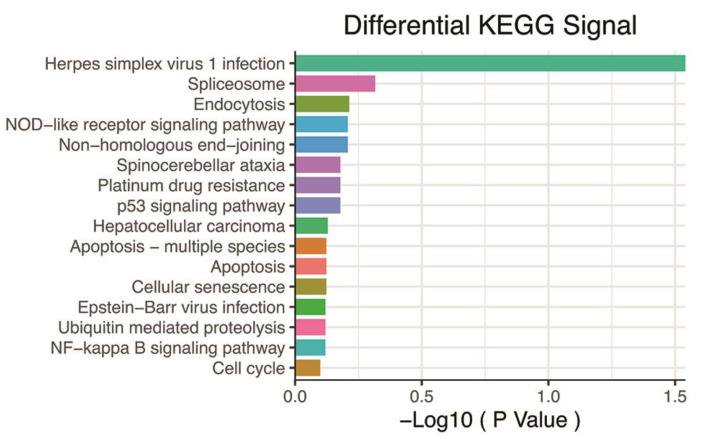
KEGG pathway analysis based on 784 genes with *p* < 0.05 and |r| > 0.6. KEGG: Kyoto Encyclopedia of Genes and Genomes.

Our correlation analysis revealed that 784 genes were related to the hypoxia score based on |r|> 0.6 and *p* < 0.05 (Supplementary Table S2). These genes were enriched in a large number of signaling pathways, such as the p53 signaling pathway, cell senescence, the NF-kappa B signaling pathway, Ubiquitin-mediated proteolysis, and apoptosis (Figure 3).

### 3.4. Immunity Analysis

**Figure 4:**
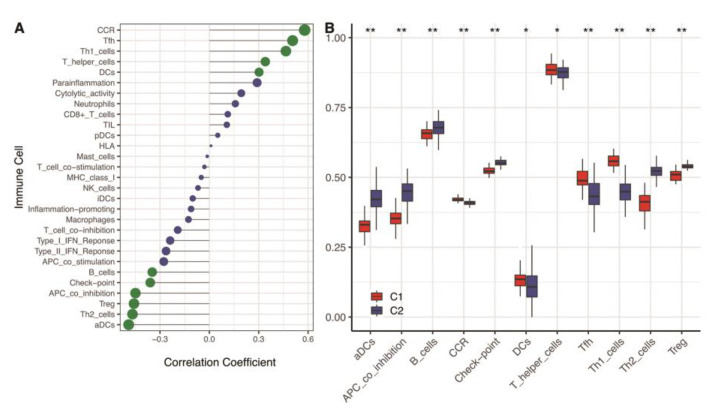
Immune cell levels of two hypoxia subtypes in the training cohort. (**A**) ssGSEA analysis based on 29 immune cellular components. (**B**) Box plots of immune cell infiltration in two subtypes. ** means *p* < 0.01; * means *p* < 0.05. ssGSEA: single-sample gene set enrichment analysis.

Our results revealed that hypoxia was significantly correlated with the immune microenvironment (Figure 4A). The C2 cluster with a low hypoxia status had a higher level of activated dendritic cells (aDCs), APC co-inhibition, B cells, Check-point cells, type-2 T helper cells (Th2), and regulatory T cells (Treg). While the C1 cluster with a higher hypoxia status had a higher level of cytokine receptor (CCR), dendritic cells (DCs), T helper cells, T follicular helper cells (Tfh), and type-1 T helper cells (Th1) (Figure 4B). That implied that the immune microenvironment difference might depend on the hypoxia status. 

### 3.5. Construction and Verification of a Novel Hypoxia-Related Marker

**Figure 5:**
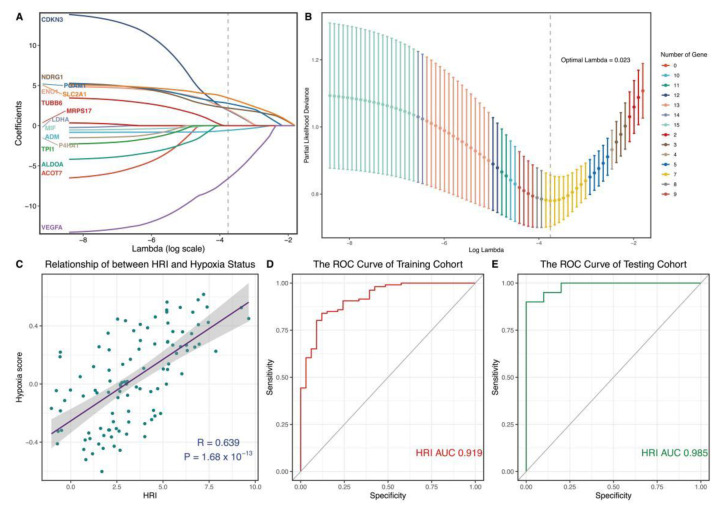
Construction and verification of hypoxia−related diagnostic marker. (**A**,**B**) Hub hypoxia-related genes were identified by LASSO regression analysis. Optimal Lambda = 0.023. (**C**) Pearson correlation analysis between HRI and hypoxia status. (**D**,**E**) Time-dependent ROC curve analysis of HRI. (**D**) Training cohort. (**E**) Testing cohort. LASSO: least absolute shrinkage and selection operator; ROC: receiver operating characteristic; HRI: hypoxia-related index.

In the above analysis, we could confirm that hypoxia is significantly correlated with the pathogenies of OA. The exploration of novel hypoxia-related markers could improve OA diagnosis in clinical settings. Therefore, we used LASSO regression analysis to select seven important hypoxia-related genes (ADM, CDKN3, ENO1, NDRG1, PGAM1, SLC2A1, and VEGFA) for constructing a novel marker (Figure 5A,B). To facilitate a clinical application, we transferred seven genes into the HRI using GLM: Our Pearson correlation analysis revealed that HRI was closely positively correlated with hypoxia status (Figure 5C, r = 0.639, *p* < 0.05). It implied that HRI could efficiently reflect the hypoxia level of OA. In the ROC curve analysis, we also found that HRI performed with high accuracy and potentiality (Figure 5D, AUC training cohort = 0.919). It was verified in an independent cohort (Figure 5E, AUC testing cohort = 0.985).

### 3.6. Identification of 12 Drugs for the Treatment of High Hypoxia Score Patients Based on CMap Analysis 

**Figure 6:**
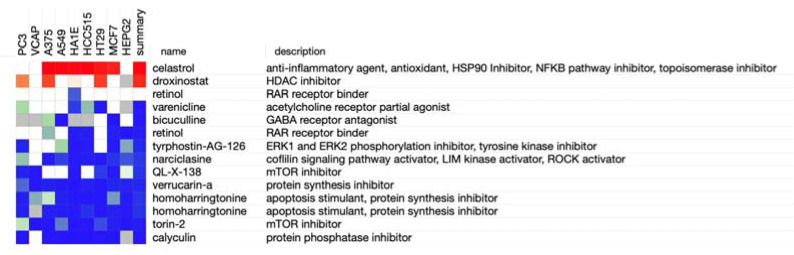
Potential therapeutic agents were identified by the CMap analysis. CMap: connectivity map.

Based on our criteria, we found 12 potential therapeutics related to the hypoxia score. These are celastrol, droxinostat, retinol, varenicline, bicuculline, tyrphostin-AG-126, narciclasine, QL-X-138, verrucarin-A, homoharringtonin, torin-2, and calyculin (Figure 6). These components may be of great value in the prevention and treatment of OA and the treatment of their symptoms.

## 4. Discussion

OA is a chronic disease characterized by an imbalance of chondrocyte anabolic and catabolic activities [30]. In recent years, it has been revealed that hypoxia plays a crucial role in cartilage metabolism and the development of OA [31]. On the one hand, hypoxia changes the phenotype of OA chondrocytes and enhances chondrocyte interaction, promotes apoptosis and the autophagy of chondrocytes, and leads to cartilage destruction [32]. On the other hand, hypoxia can affect the inflammatory microenvironment and innate or adaptive immunity to promote OA joint pain and cartilage damage [33,34]. In this study, we found that many hypoxia-related genes and hypoxia status exhibited significant values concerning OA, which is consistent with previous studies [15,35].

The relationship between hypoxia and OA is complex. In the functional analysis, we found that hypoxia is involved in multiple pathways in OA development. P53 plays an important role in apoptosis, the inhibition of growth, the inhibition of cell cycle progression, and senescence after cell stress [36]. Under hypoxia, p53 inhibits reactive oxygen species (ROS) generation and ROS detoxification to promote cell survival by promoting cell metabolic energy generation in OA [37]. Apoptosis is the programmed death of most cells under hypoxia. Studies have revealed that apoptosis leading to chondrocyte loss is one of the mechanisms of cartilage degeneration [38]. NF-κB is regarded as a transcriptional factor that is activated by several pro-inflammatory cytokines. It is closely related to inflammation, apoptosis, oxidative stress, and the extracellular matrix degradation of chondrocytes [39]. The ubiquitin–proteasome pathway is a major proteolytic pathway. It plays a key role in regulating cell proliferation, survival, and differentiation, and its destruction leads to many human diseases. Increased ubiquitination in OA knees can lead to proteasome damage and chondrocyte apoptosis [40].

In general, OA is classified as non-inflammatory arthritis and is predominantly a degenerative disease of old age [27]. However, with further study of OA, immunity is considered to be an important part of OA pathogenesis [41,42]. Except for multiple signaling pathways, our results found that hypoxia was correlated with multiple immune cells in OA. CCR may regulate the expression of MMP-3 to reduce the storage of proteoglycan and thus participate in the degradation of OA cartilage [43]. DCs secrete inflammatory cytokines with peripheral tolerance potential, which will significantly promote chondrogenesis and reduce inflammation [44]. It has potential immunotherapeutic value in OA. B cell infiltration is directly associated with the severity of local inflammation [45]. Th1, Th2, Tfh, and Treg cells are the main T-cell subsets associated with OA pathology, which may be the markers of OA disease activity [46,47,48]. In this study, high hypoxia status had higher levels of CCR, Tfh, and Th1. Low hypoxia status had higher levels of aDCs, Th2, and Treg. It suggests that different immune cell infiltrates may affect the hypoxia environment, which plays an essential role in the occurrence and development of OA.

Hence, we believed that hypoxia could improve the accuracy of the diagnosis of OA. In this study, we found a novel marker that is composed of seven hypoxia-related genes (ADM, CDKN3, ENO1, NDRG1, PGAM1, SLC2A1, and VEGFA), which are optimal signatures for the diagnosis of OA. HRI was construed to be a marker for the diagnosis and prognosis of OA. ADM, an anti-apoptotic peptide, could promote synovial cell apoptosis and chondrocyte dedifferentiation in inflammatory arthritis by increasing the production of oxidative stress and pro-inflammatory cytokines [49,50]. CDKN3 is an important regulator of the cell cycle. CDKN3 downregulation is involved in the formation of a hypoxic microenvironment by inhibiting the proliferation and invasion of cancer cells [51]. ENO1, a metabolic enzyme involved in pyruvate synthesis, is upregulated under hypoxic conditions. It is involved in the transformation of oxidative decomposition into glycolysis and lactic acid formation [52]. NDRG1 is a potent metastatic repressor [53]. Dong et al. showed that NDRG1 silencing significantly induced apoptosis under hypoxia conditions and allowed mitochondrial damage to be induced and disrupt hypoxia-enhanced aerobic glycolysis [54]. That agreed with the pathological features of OA. PGAM1, a glycolytic enzyme, can activate inflammatory cytokines and induce chondrocyte apoptosis in OA [55]. SLC2A1 is a metastable glucose transporter and is related to the normal proliferation of the growth plate chondrocytes [56]. VEGFA is an important regulator of cell, bone, and angiogenesis [57]. The overexpression of VEGFA promoted cell proliferation, inhibited apoptosis, and reduced matrix degradation in OA chondrocytes [58]. Above all, the novel marker showed great potential for clinical applications, which provides new insights into the search for effective targets and drugs for the treatment of OA.

The current treatment of OA is only for symptoms but cannot prevent or cure OA [28]. Discovering effective and sensitive drugs for OA helps improve the patient’s quality of life. The CMap analysis showed that 12 drugs were identified as treatment options for OA. Celastrol, a naturally extracted compound with anti-inflammatory and antioxidant properties, also acts as an HSP90 [59], NF-κB pathway [60], and topoisomerase inhibitor [61]. It plays a strong capacity in anti-inflammation. Droxinostat, a Histone Deacetylase inhibitor, increases intracellular oxidative stress and induces cell apoptosis [62]. Torin-2, a novel mammalian target of the rapamycin inhibitor, can activate autophagy by inhibiting the negatively-regulated PI3K/Akt/mTOR signaling pathway [63]. Narciclasine, a natural compound of Haemanthus coccineus L belonging to the Amaryllidaceae family, exhibits strong anti-inflammatory activity and attenuates the production of ROS [64]. According to current drug experiments, we implied that these components have a potential value in the treatment of OA, but the remaining other drugs have not been reported, and further exploration of these drugs is conducive to improving the current treatment status of OA.

The mechanism of hypoxia and OA is complex and is greatly significant in the exploration and treatment of OA. There are some limitations to our study, such as the use of animal experiments, clinical validation cohorts, and experimental studies on OA are severely lacking. Thus, more evidence and experiments are needed to determine the mechanism of hypoxia, and a well-designed clinical trial is necessary to validate our marker further. 

## 5. Conclusions

In this study, we preliminary find that hypoxia plays an important role in OA. Hypoxia may involve multiple pathways and the immune environment. HRI based on seven important hypoxia-related genes (ADM, CDKN3, ENO1, NDRG1, PGAM1, SLC2A1, and VEGFA) can serve to improve diagnosis in the early and asymptomatic stages of OA. Celastrol, droxinostat, torin-2, and narciclasine may be potential compounds for the treatment of OA. Our results are useful for clinical decision-making and intervention measures.

## Figures and Tables

**Table 1:** GEO datasets.

Geo Datasets	Platform	Annotation	Sample Size
GSE48556	GPL6947	illuminaHumanv3.db	106 OA and 33 Normal
GSE55235	GPL96	hgu133a.db	10 OA and 10 Normal
GSE55457	GPL96	hgu133a.db	10 OA and 10 Normal

## Data Availability

All data were downloaded from a public database. R 4.0.4 (http://www.r-project.org/) (accessed on 25 March 2022) is an open-source software. The raw data of OA were downloaded from Gene Expression Omnibus (http://www.ncbi.nlm.nih.gov/geo/) (accessed on 25 March 2022) using the GEOquery R package, including GSE48556, GSE55235, and GSE55457.

## Supplementary Materials

The following supporting information can be downloaded at: https://www.mdpi.com/article/10.3390/genes13091501/s1, Table S1: 29 immune gene sets; Table S2: 784 hypoxia score related genes.

## Abbreviations

OA, osteoarthritis; GEO, Gene Expression Omnibus; GSVA, gene set variation analysis; LASSO, least absolute shrinkage and selection operator; HRI, hypoxia-related index; ROC, receiver operating characteristic; AUC, Area under the curve; PCA, principal component analysis; ssGSEA, single-sample gene set enrichment analysis; KEGG, Kyoto Encyclopedia of Genes and Genomes; CMap, Connectivity Map.
